# Autocatalytic association of proteins by covalent bond formation: a Bio Molecular Welding toolbox derived from a bacterial adhesin

**DOI:** 10.1038/srep43564

**Published:** 2017-03-02

**Authors:** J. Bonnet, J. Cartannaz, G. Tourcier, C. Contreras-Martel, J. P. Kleman, C. Morlot, T. Vernet, A. M. Di Guilmi

**Affiliations:** 1 Institut de Biologie Structurale (IBS), Univ. Grenoble Alpes, CEA, CNRS, 38044 Grenoble, France; 2 Institut de Biosciences et Biotechnologies de Grenoble (BIG), Univ. Grenoble Alpes, CEA, CNRS, 38044 Grenoble, France

## Abstract

Unusual intramolecular cross-links present in adhesins from Gram-positive bacteria have been used to develop a generic process amenable to biotechnology applications. Based on the crystal structure of RrgA, the *Streptococcus pneumoniae* pilus adhesin, we provide evidence that two engineered protein fragments retain their ability to associate covalently with high specificity, *in vivo* and *in vitro*, once isolated from the parent protein. We determined the optimal conditions for the assembly of the complex and we solved its crystal structure at 2 Å. Furthermore, we demonstrate biotechnological applications related to antibody production, nanoassembly and cell-surface labeling based on this process we named Bio Molecular Welding.

Cross-linking of amino acid side chains is an efficient way to promote protein folding and stabilization at the post-translational level. The most common feature is the formation of intra- or intermolecular disulfide bonds to stabilize tertiary and quaternary structures, respectively. One prominent example is given by antibody molecules, whose disulfide-bond mediated stability in blood circulation is crucial for efficient function. Interactions of Gram-positive bacteria with host organisms are resolved by adhesins like pilins and MSCRAMMs (microbial surface components recognizing adhesive matrix molecules) in which unusual intramolecular cross-links have been recently discovered^1^,^2^,^3^. As adhesins are surface-exposed proteins, they must resist various antibacterial defense mechanisms like degradation by enzymes (proteases, peroxidases) or unfolding upon pH changes. The covalent linkages present in adhesins enhance their thermal stability and resistance to proteases degradation, thus stabilizing their tertiary structure^4^,^5^,^6^,^7^,^8^. In the case of pili, the presence of these isopeptide bonds, together with the fact that pilins are covalently linked to each other, make Gram-positive pili highly resistant to mechanical stress and degradation^9^.

Pilins and MSCRAMMs share a common topology: a N-terminal signal peptide followed by extracellular domain(s), a cell wall sorting motif (Leu-Pro-x-Thr-Gly or a variant) recognized by sortases which covalently anchor proteins to peptidoglycan, a transmembrane helix and a short cytoplasmic sequence. Most pilins and MSCRAMMs have an elongated shape formed by the association of domains, which resemble Ig-like β-sandwich domains^2^. Intramolecular isopeptide bonds are mostly formed between the side chains of Lys and Asn, although Lys-Asp bonds also exist^5^,^10^. These residues are positioned in an hydrophobic core in which the pKa of the Lys is reduced, allowing nucleophilic attack of the Cδ atom of either Asn or Asp, followed by a proton shuttle involving an adjacent Glu or Asp^5^,^10^,^11^. Although not involved in protein stabilization, thioester bonds established between Cys and Gln residues have also been described^5^. The last intramolecular bond reported is an ester bond formed between a Thr and a Gln side chain in a putative *Clostridium perfringens* MSCRAMMs, through an autocatalytic mechanism that differs from that of Lys-Asn/Asp bonds^1^.

Intramolecular isopeptide cross-linking is autocatalytic. Indeed, there is neither need for a chaperone-assisted machinery like for disulfide bond formation, nor assistance by dedicated enzymes like transglutaminase, ubiquitinase or sortase. In addition, no specific oxido-reduction environment is required. These attractive features have been exploited in biotechnology through the development of peptide tags^12^,^13^. This pioneering strategy developed by the Howarth’s group consists in splitting a pilin into two separate protein-peptide pairs and reconstituting specifically the globular domain through the covalent association between the two partners. This protein-peptide tag system has been fused to various proteins-of-interest for several applications, including polymerization of linear, branched or combinatorial polyproteins via iterative sequential reactions on solid-phase^14^, design of new protein architectures^15^, protein cyclization to resist unfolding^16^ and vaccination^17^.

Based on the crystal structure of RrgA, the pilus adhesin from *Streptococcus pneumoniae*, we exploited a unique protein-protein fusion system. We provide evidence that two engineered protein fragments from RrgA retain their ability to associate covalently *in vivo* and *in vitro* although being isolated from the parent protein. We report the biochemical and structural characterization of the complex assembly and validate several biotechnological applications based on this process, that we called Bio Molecular Welding (BMW).

## Results and Discussion

### Covalent and spontaneous association between Jo and In partners

**Figure 1:**
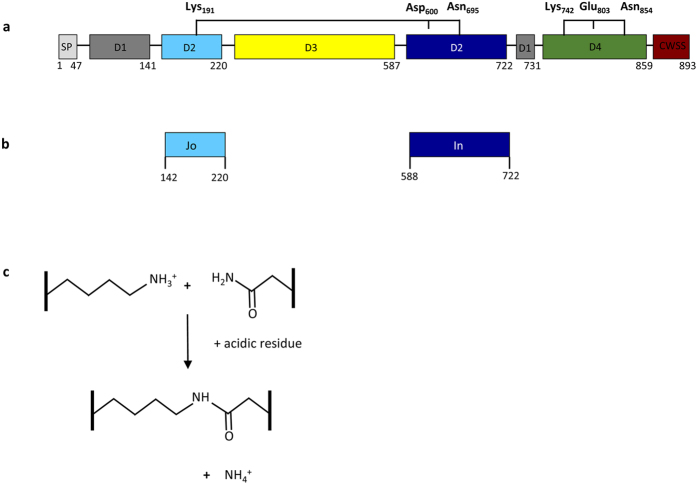
Intramolecular isopeptide bonds in RrgA domains. (**a**) Schematic topology of RrgA composed of four domains (D1 to D4) in addition to the peptide signal (SP) and the cell wall sorting signal (CWSS). D3 is inserted into D2, and D2/D3 are inserted into D1. D2 and D4 are stabilized by intramolecular isopeptide bonds figured by brackets. In D2, the isopeptide bond between the side chains of Lys_191_ and Asn_695_ is stabilized by Asp_600_. The isopeptide bond in D4 between Lys_742_ and Asn_854_ is stabilized by Glu_803_. D2 is formed by the association of two distinct regions, hereafter called Jo and In, held together by the isopeptide bond. (**b**) Jo and In constructs. (**c**) The amide bond formation between Lys and Asn side chains depends on an acidic residue, either Glu or Asp.

RrgA is the structural pilin positioned at the tip of *S. pneumoniae* pilus-1 and contains four unusual elongated domains^8^. Domains D1 and D2 carry secondary structure elements, from both the N-terminal and C-terminal RrgA regions, into which other sequences have been inserted (domain D3 is inserted into D2; D2/D3 are inserted into D1) (Fig. 1a). RrgA harbors two intramolecular isopeptide bonds, one located in the D2 domain, and the second in D4. The D4 domain (Fig. 1a) precedes the C-terminal CWSS sorting motif and its fold is highly reminiscent of IgG domains. The isopeptide bond stabilizing the D4 domain is formed by Lys_742_ and Asn_854_, the hydrogen bonding stabilization being provided by Glu_803_^8^. The D2 domain is formed by the association of 2 “halves” encoded by sequences flanking the D3 domain (Fig. 1a). The distinct N-(residues 144–220) and C-terminal (residues 593–722) sub-domains, hereafter referred as Jo and In, respectively (Fig. 1b), clasp into an 11-β stranded sandwich locked by an isopeptide bond between the side chains of Lys_191_ in Jo and Asn_695_ in In (Fig. 1a)^8^. The amide bond formation between the Lys and the Asn side chains is dependent on Asp_600_^8^ (Fig. 1c).

**Figure 2:**
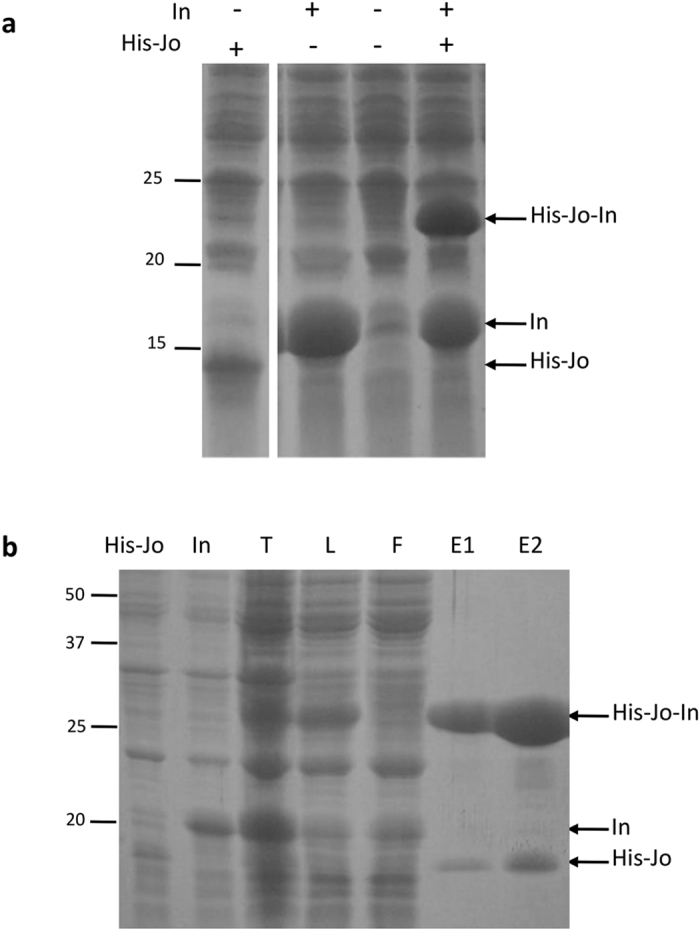
Jo and In covalently associate in bacterial crude extracts. (**a**) Crude extracts from *E. coli* cells expressing His-Jo and/or In proteins. Co-expression of His-Jo and In leads to the formation of the covalent His-Jo-In complex. (**b**) Reconstitution of the covalent His-Jo-In complex by mixing separate *E. coli* cultures expressing either His-Jo or In. The His-Jo-In complex was purified by Ni-affinity chromatography. His-Jo and In: total cell extract of separated cultures, T: total cell extract of mixed cultures, L: load, F: flow through, E1 and E2: elution fractions containing the His-Jo-In complex. The polyacrylamide gels were run in denaturanting conditions (SDS-PAGE) and stained by Coomassie blue. The molecular mass standards (in kDa) are indicated on the left.

To test whether Jo and In sub-domains retain their ability to covalently interact and reconstitute the RrgA D2 domain, DNA sequences encoding His-Jo and In were cloned into pETDuet plasmid under the control of a T7 promoter for co-expression in *Escherichia coli* cytoplasm (Fig. 2a and S1). We did not notice any effect of the properties of the *E. coli* cultures, such as optical density, on the level of Jo and In association. As observed in crude *E. coli* lysates when expressed individually, His-Jo and In sub-domains are produced as soluble proteins and the molecular masses observed by SDS-PAGE are consistent with the calculated molecular mass values: 10 470 Da and 15 091 Da, respectively (Fig. 2a). Co-expression of His-Jo and In was induced with IPTG for 3 h at 37 °C, a period long enough to allow Jo and In association. Indeed, when His-Jo and In were co-expressed, an additional species migrating at about 22 kDa is observed, indicating that the covalent complex between His-Jo and In has formed with the expected 1:1 stoichiometry altough free In is still detected, likely due to overexpression of In over His-Jo (Fig. 2a). Observation of the recombinant His-Jo-In complex in *E. coli* cell extracts provides the first evidence that isolated Jo and In sub-domains are able to covalently associate with high efficiency and specificity in the crowded molecular environment of bacterial cytoplasm.

We next tested whether His-Jo and In, produced separately, would associate *in vitro*. Cultures expressing either His-Jo or In were mixed after the induction period and before sonication. The total contact time, starting when cultures were mixed and ending when the complex was eluted from the affinity resin is about 1 h, and the whole experiment was carried out at 20 °C (Fig. 2b). The covalent His-Jo-In complex was observed in the crude cell extract and in the soluble fraction after centrifugation, indicating that the Jo-In association can happen after complete synthesis of the polypeptide chains (Fig. 2b, lanes T and L). The complex His-Jo-In was efficiently purified by Ni-affinity chromatography (Fig. 2b, lanes E1 and E2). Small quantities of His-Jo were also eluted from the Ni-column, either because an excess of His-Jo was produced over In or owing to a fraction of His-Jo unable to associate to In. Altogether, these data show that Jo and In sub-domains retain their ability to form a stable complex although isolated from the parent RrgA protein.

### Characterization of the Jo-In complex formation

**Figure 3:**
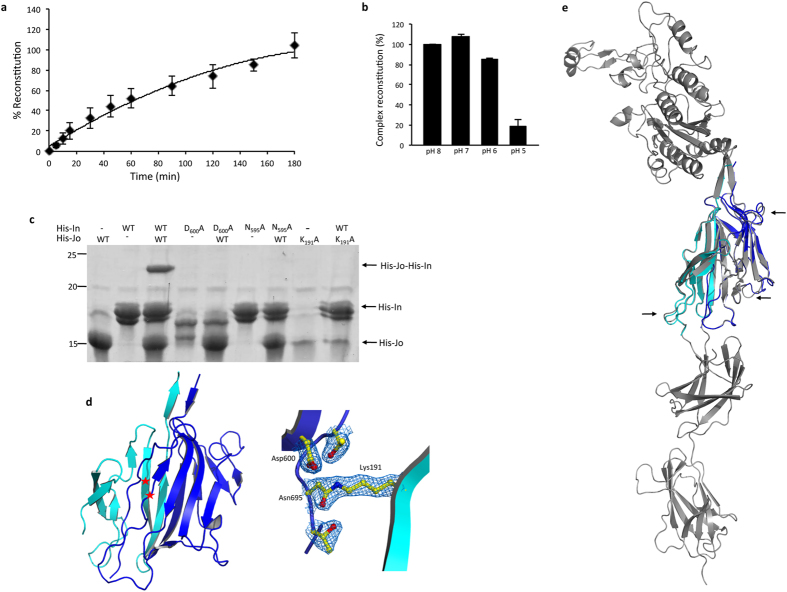
Characterization of the Jo-In complex. (**a**) Kinetics of complex formation. A quantity of 10 μM of purified His-Jo and His-In were mixed and incubated at 20 °C for different durations before analysis by Coomassie blue-stained SDS-PAGE. The amount of His-Jo and His-Jo-His-In complex in the mix was quantified using ImageJ and the calculated proportion of the complex (ratio complex: Jo) was plotted against time (min) (see Methods). Error bars represent standard deviation of three independent experiments. (**b**) pH dependency of complex formation. Jo and In partners were mixed at 25 μM in buffered solutions at pH 5 to 8 and incubated at 20 °C for 60 min. The proportion of the complex was calculated, reported for each pH value and normalized to 100%. Error bars represent standard deviation of two independent experiments. (**c**) The covalent complex is observed in the presence of the wild-type (WT) proteins and is impaired by Lys_191_Ala, Asn_695_Ala and Asp_600_Ala mutations. For clarity reason, the one-letter amino acid code is figured. Wild-type and mutant forms of purified His-Jo and His-In were incubated for 30 min at 20 °C. The samples were analyzed by Coomassie blue-stained gels. (**d**) Ribbon representation of the His-Jo-In complex (left panel). Jo is colored in cyan and In in blue. Red asterisks indicates the position of Lys_191_ in Jo and Asn_695_ in In proteins. Right panel: close-up view of the key residues for the amide bond formation (represented in sticks), the electron density is also shown. (**e**) Crystal structure of the Jo-In complex (cyan and blue, respectively) superimposed on the D2 domain (grey) in the context of the full-length RrgA protein (PDB ID: 1WW8). Arrows indicate minor differences in the structures superposition.

To characterize in *in vitro* conditions the covalent association of Jo and In proteins, the complex was reconstituted from independently purified His-Jo and His-In proteins and its assembly was monitored over time (see Methods). The complex was detected upon 5 min of incubation, about 60% of the complex was reconstituted after 90 min and about 100% after 180 min (Fig. 3a).

The presence of a negatively charged residue (Asp_600_) in the vicinity of residues involved in the isopeptide bond led us to test the influence of the pH in the autocatalytic reaction. The reaction was efficient at pH 8 and pH 7 since 100% of the complex was reconstituted after 1 h, the formation of the complex was slightly reduced to 90% at pH 6 and largely inefficient at pH 5 since only 18% of the complex could be detected (Fig. 3b). This pH dependency suggests that a residue, likely Asp_600_, with a pKa value between 5 and 6 might need to be deprotonated to participate in the reaction mechanism.

The covalent association of Jo and In was confirmed by measuring the molecular mass of the complex by electro-spray ionization mass spectrometry (ESI-MS) as reported previously^7^. The measured molecular mass of 26 772 Da is in accordance with the predicted mass assuming the formation of the isopeptide bond, which results in the loss of a NH_3_ group of 17 Da (Table 1)^8^.

To further demonstrate that Jo and In reconstitute the RrgA D2 domain in a spontaneous and covalent manner, the residues involved in the isopeptide bond, Lys_191_, Asp_600_ and Asn_695_ were individually mutated into Ala^8^ and the formation of the complex was tested. Wild-type (WT) and variants of Jo and In were purified independently and mixed in different combinations (Fig. 3c). The covalent complex His-Jo-His-In was formed only when both WT forms of Jo and In were mixed, while each point mutation totally impaired the complex assembly (Fig. 3c).

The rather slow kinetics of the Jo-In complex formation might originate from an alteration of the structure of the protein fragments when isolated from the parent RrgA protein. In this case, one might expect the His-Jo-In complex to display a structure slightly different from the D2 domain of RrgA. To test this possibility, we have solved and refined the crystal structure of the His-Jo-In complex (PDB: 5MKC) at 2.04 Å (Table 2). The structure of the complex shows that the Jo and In moieties grasp into an 11-β-stranded sandwich (Fig. 3d). The two partners are locked together by the isopeptide bond formed by the fusion of the side chains of Lys_191_ and Asn_695_ in proximity to Asp_600_ (Fig. 3d). The structure of the complex is indistinguishable from that of the RrgA D2 domain (rms of 0.61 Å), indicating that the fold adopted by the Jo and In partners produced individually, although possibly different from that acquired in the context of the full RrgA sequence, still remains compatible with the complex formation (Fig. 3e). Parental RrgA and Jo-In complex structures were superimposed and neither significant differences nor flexibility were detected despite slight variations observed in a few loops (depicted in Fig. 3e by arrows).

### Application 1 of the BMW^JOIN^ process: antigen display

**Figure 4:**
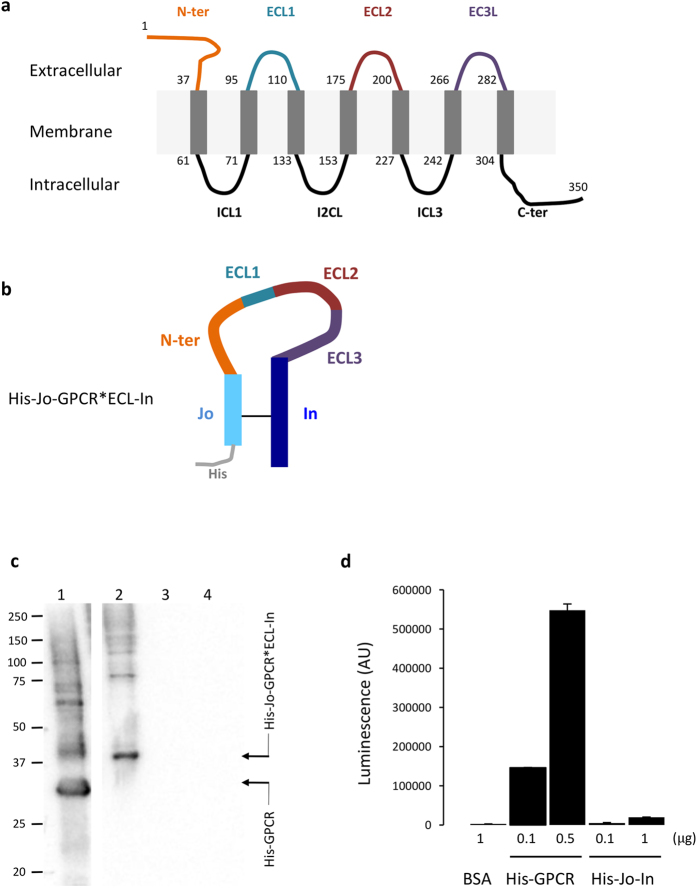
The BMW^JOIN^ toolbox can be used for presentation of protein loops and antibody production. (**a**) Topology of the membrane protein used in this study, a GPCR with seven transmembrane helices. ECL: extracellular loop, ICL: intracellular loop. The residues delimiting each transmembrane helix (grey rectangles) are indicated. (**b**) Concatenation of the extracellular regions of the GPCR (G-protein coupled receptor) and fusion to Jo and In. The so-called purified His-Jo-GPCR * ECL-In (*refers to the soluble regions of the GPCR protein) was used to immunize rabbits. (**c**) Western Blot performed with anti-GPCR * ECL purified antibodies. 1: His-GPCR; 2: His-Jo-GPCR * ECL-In; 3: His-Jo-In; 4: Hla-His (hemolysin alpha of 35 kDa used as a negative control). The molecular mass standards (kDa) are indicated on the left. (**d**) ELISA assay performed with anti-GPCR * ECL purified antibodies. Coated quantities of His-GPCR, His-Jo-In and BSA are indicated. Error bars represent standard deviation of duplicates and the results shown are representative of two independent experiments.

The BMW^JOIN^ platform has the following attractive features: (1), His-Jo-In is expressed in *E. coli* to high levels (about 10 mg/L culture); (2), the complex is highly soluble; (3), fusion of the Jo and In halves into a single polypeptide chain should result in the production of a circular protein upon formation of the isopeptide bond, which might displays a potentially enhanced stability as previously shown^16^. To test this latter possibility, Jo and In were joined together with an intervening 7-residues peptide linker and this construct was expressed at high level in *E. coli* (about 15 mg/L culture). The purified product, His-Jo-GSTPGSV-In, folds into a circular single polypeptide chain containing the covalent isopeptide bond as confirmed by ESI-MS (Table 1). The fact that the Jo-In association was not impaired by the presence of a heterologous peptide prompted us to exploit this property as a platform to expose otherwise unstable protein sequences. As a first application, we employed this tool to produce protein fragments to be used as antigens for the production of antibodies against difficult-to-express proteins, in particular transmembrane helices. We selected a membrane protein from the GPCR (G-protein coupled receptor) family displaying 7 transmembrane anchors, 3 intracellular and 3 extracellular loops; the N- and C-terminal tails being exposed extracellularly and intracellularly, respectively (Fig. 4a). The sequences coding for all the extracellular regions were fused in an artificial sequence and inserted between Jo and In, leading to a His-Jo-GPCR * ECL-In construct (Fig. 4b).

Rabbit polyclonal serum was generated against the His-Jo-GPCR * ECL-In (His-Jo-G-protein coupled receptor * extracellular-In) purified circular protein. Immuno-purifications of sera were performed in order to remove antibodies against His-tag, Jo and In proteins. A first purification step using a His-Jo-In-coupled resin efficiently removed anti-His-Jo-In IgG present in the serum. Indeed, the immuno-purified antibodies did not recognize neither the His-Jo-In complex nor the His-tagged hemolysin alpha (Hla-His) protein used a control (Fig. 4c, lanes 3 and 4) but they allowed efficient detection of the His-Jo-GPCR * ECL-In construct, the migration of which is in accordance with the expected 33 238.90 Da molecular mass (Fig. 4c, lane 2). More interestingly, they also enabled efficient detection of a full-length His-GPCR construct produced in a cell-free system (Fig. 4c, lane 1). The His-GPCR protein migrates at about 30 kDa, a value that is lower than the calculated mass of 41 928.30 Da. Such discrepancy in electrophoretic migration is often observed in multi-membrane proteins due to detergent binding^18^. The upper bands likely correspond to non-covalent multimerized forms of the protein (Fig. 4c, lane 1). The data obtained by Western blot were further supported by ELISA assay using the purified serum (Fig. 4d). In this assay, a negligible luminescence signal was obtained when the purified serum was incubated with the His-Jo-In complex while dose-dependent signal was detected when the purified serum was incubated with the full-length His-GPCR construct.

In conclusion, our procedure, that we called Loop-Of-Loops (LOL), provides a fast and inexpensive way to deliver ELISA and Western blot-compatible rabbit polyclonal antibodies derived from the exposed regions of membrane proteins without the need to produce and purify the full-size parent protein or to synthesize expensive and often poorly antigenic peptides. Five different projects were set up with the same procedure: antibodies against two other GPCRs as well as a potassium channel were successful in delivering antibodies. Only one failed.

### Application 2 of the BMW^JOIN^ process: large molecular assemblies

**Figure 5:**
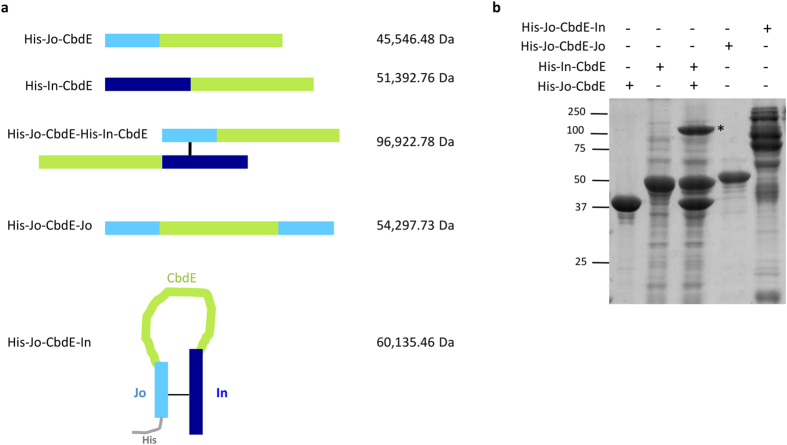
Supra-molecular assembly of BMW^JOIN^-fusion proteins. (**a**) Topology of CbdE constructs fused to Jo and/or In in various configurations. The masses of the purified proteins, measured by ESI-MS, are indicated on the right. (**b**) A covalent complex is formed between His-Jo-CbdE and His-In-CbdE (indicated by a star). His-Jo-CbdE-Jo appears as a single species while His-Jo-CbdE-In generates a wide range of high-molecular species. The assay used purified His-Jo-CbdE, His-Jo-CbdE-Jo and His-Jo-CbdE-In and a total cell extract for His-In-CbdE to test the specificity of the interaction.

Veggiani *et al*.^14^ have reported a system derived from the D4 Ig-like domain of *S. pneumoniae* RrgA for the programmable and efficient construction of polyproteins. These artificial structures can have various architectures not restricted to linear forms but also allowing for the design and synthesis of branched and combinatorial proteins. We have explored the possibility to use the BMW process (derived from the non Ig-like D2 domain of *S. pneumoniae* RrgA) to construct large molecular protein assemblies. The circularization of Jo and In expressed as a single polypeptide chain while separated by a flexible linker is a first step toward using the BMW^JOIN^ platform for the production of novel structures. The insertion between Jo and In of sequences of various sizes and properties might allow the production of reagents able to autoassemble in a controlled fashion. To explore this possibility, we have selected Choline-Binding Domain E (CbdE), a 292 residues-long protein of moderate flexibility^19^ from *S. pneumoniae*. This surface-exposed protein, containing a repetition of 10 modules was used as a spacer between Jo and In (Fig. 5a). All constructs were His-tagged and purified by Ni-affinity chromatography prior to *in vitro* mixing. SDS-PAGE migration patterns and ESI-MS measurements indicated that His-Jo-CbdE, His-In-CbdE and His-Jo-CbdE-Jo remain monomeric (Fig. 5a and b). Co-expression of His-Jo-CbdE with His-In-CbdE led to the production of both monomeric proteins together with a species with molecular mass determined by ESI-MS which corresponds to the isopeptide bond-linked complex (Fig. 5a and b). The expression of His-Jo-CbdE-In resulted in the formation of various combinations of Jo-In complexes (Fig. 5b). When CbdE was fused to both Jo and In proteins, a variety of Jo and In associations occurred *in vivo* leading to different species of high molecular weight as observed after protein purification (Fig. 5b last lane). In addition, a monomeric form of His-Jo-CbdE-In of 60 135 Da was detected by ESI-MS in which Jo and In were linked through the isopeptide bond, leading to a circular molecule as depicted in Fig. 5a.

These data indicate that stable high molecular structures can be obtained from the insertion of a large protein sequence fused in between Jo and In. Further work is in progress to control the design and production of new BMW-derived network of proteins to tailor large protein assemblies that would complete the tools developed from the Ig-like D4 domain^14^.

### Application 3 of the BMW^JOIN^ process: detection of cell surface-exposed proteins

The presence of proteins exposed at the cell surface is usually performed by immunodetection after subcellular fractionation or limited proteolytic cleavage and alternatively by an *in vivo* approach related to the measurement of the extracellular activity of an enzyme (alkaline phosphatase, β-lactamase) fused to the protein of interest. Altogether, these techniques are time-consuming, subject to contaminations and to artefacts. In this work, we tested whether the BMW^JOIN^ tool would be more efficient to address these issues. The Choline-binding-protein (CbpE) from *S. pneumoniae* was chosen as a model of secreted protein associated to the bacterial cell wall. CbpE contains a signal peptide, a N-terminal phosphorylcholine esterase catalytic domain, followed by the CbdE region interacting non-covalently with choline residues decorating the teichoic acids in *S. pneumoniae*.

**Figure 6:**
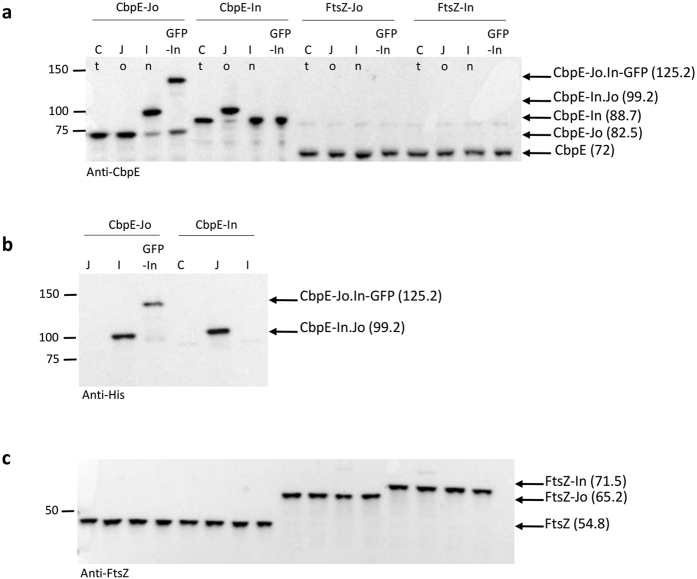
The BMW^JOIN^ toolbox can be used for detection of surface-exposed proteins. (**a**) Immunoblots analysis of whole cell lysates from unencapsulated pneumococcal cells expressing CbpE-Jo, CbpE-In, FtsZ-Jo and FtsZ-In and incubated with PBS (Ct), His-Jo (Jo), His-In (In) or His-GFP-In (GFP-In) with anti-CbpE serum. (**b**) Immunoblots analysis of samples shown in (A) with anti-His-tag. (**c**) Immunoblots analysis of samples shown in (A) with anti-FtsZ serum. The CbpE and FtsZ variants and their calculated molecular masses (kDa) are indicated on the right side of the panels and the molecular masses standards (kDa) are marked on the left.

The 3′ end of the *cbpE* gene was fused to the sequences encoding either Jo or In, or FtsZ, a cytoplasmic protein used as a negative control in the unencapsulated pneumococcal strain R800. We first tested whether Jo and In could recognize and associate to their partners when they were fused to CbpE. Pneumococcal cultures expressing the various fusion proteins were harvested at exponential growth phase, washed and incubated with purified His-Jo, His-In or His-GFP-In proteins for 1 h at 20 °C (GFP accounts for Green Fluorescent Protein). Cells were washed and comparable amounts of material were loaded on SDS-PAGE for immunodetection using anti-CbpE or anti-His antibodies. Protein species with apparent molecular masses of about 100 kDa were detected when cells that express CbpE-Jo (82.5 kDa) or CbpE-In (88.7 kDa) were incubated with His-In and His-Jo, respectively (Fig. 6a and b). In a similar way, incubation of His-GFP-In with cells expressing CbpE-Jo led to the formation of a protein species migrating just below the 150 kDa molecular mass marker. The about 100 kDa species likely correspond to the CbpE-Jo-In complex (theoretical molecular mass of 99.2 kDa) while the <150 kDa species is consistent with the CbpE-Jo-In-GFP complex (theoretical molecular mass of 125.2 kDa) (Fig. 6a and b). The resistance of these large protein species to SDS-PAGE denaturing conditions indicates that they result from the covalent association of the Jo and In partners. Neither incubation of the cells with buffer nor with the same BMW^JOIN^ partner (*i.e*. CbpE-Jo plus His-Jo or CbpE-In plus His-In) led to a mass shift, confirming the specificity of the Jo-In interaction.

To verify that cytoplasmic proteins were not accessible to exogenously added Jo and In proteins, similar experiments were performed with cells expressing Jo and In fusions to the cytoplasmic division protein FtsZ. The apparent masses of the different variants of FtsZ are in accordance with the expected values: FtsZ (54.8 kDa), FtsZ-Jo (65.2 kDa) and FtsZ-In (71.5 kDa) (Fig. 6c). No mass increase of FtsZ was observed whatever the BMW^JOIN^ combination tested, demonstrating that the exogenous BMW^JOIN^ proteins do not penetrate into the cytoplasm to reach FtsZ- BMW^JOIN^ proteins (Fig. 6c). These results indicate that only proteins exposed at the cell surface and bearing one BMW^JOIN^ partner are accessible to the complementary ligand.

In conclusion, this method aimed to detect proteins exposed at the cell surface based on the Jo-In association is rapid, little sample processing is required since whole live cells are used. Altogether, these results open the way to exploit the BMW^JOIN^ toolbox to detect proteins exposed at the bacterial cell surface, even in the absence of specific antibody. Work is in progress to apply this tool to Gram-negative bacteria.

### Application 4 of the BMW^JOIN^ process: localization of proteins exposed at the surface of live cells

Labeling the surface of live eukaryotic or prokaryotic cells is a challenging issue. The most common way is to genetically fuse the protein-of-interest to fluorescent proteins. The formation of the GFP chromophore, which arises from the spontaneous cyclization of a tripeptide is a post-translational modification dependent on the properly folded GFP scaffold^20^ which is impaired after secretion^21^. To circumvent this problem, the superfolder GFP (sfGFP) variant was engineered to display more robust refolding properties^22^,^23^. sfGFP was successfully used to detect fusion proteins exported in the periplasm of *E. coli*^22^,^24^ but not yet employed to address the localization of secreted protein exposed at the surface of Gram-positive bacteria.

**Figure 7:**
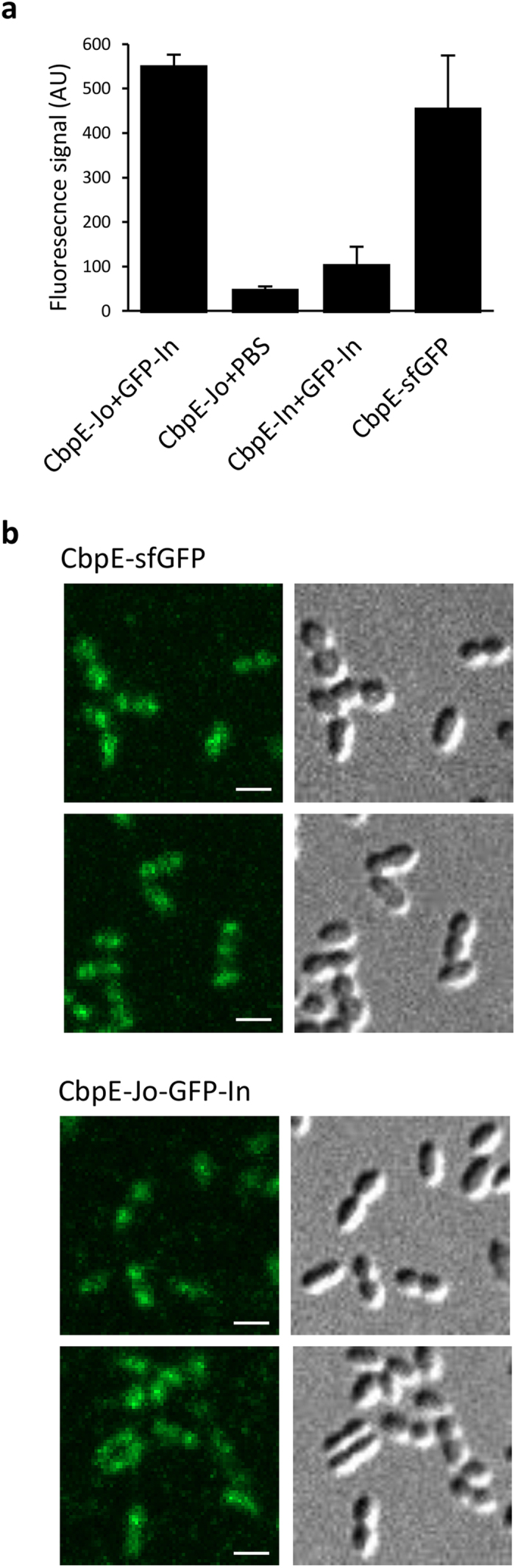
The BMW^JOIN^ toolbox can be used for localization of surface-exposed proteins. (**a**) Quantification of the fluorescence signal (see Methods) observed by confocal fluorescence microscopy of unencapsulated pneumococcal cells expressing CbpE-Jo or CbpE-In incubated with PBS or GFP-In. Cells expressing CbpE-sfGFP were also observed in the same conditions. The error bars correspond to the standard deviation of the analysis of five independent frames acquired for each sample. (**b**) Live *S. pneumoniae* cells expressing CbpE-sfGFP (upper panels) or CbpE-Jo (lower panels) complexed to GFP-In were visualized during exponential phase. GFP green fluorescence (left) and DIC (Differential Interference Contrast) images (right) are shown. Scale bars: 1 μm.

Having established the formation of specific covalent Jo-In complexes at the surface of the pneumococcus, we have developed an assay for the detection of proteins at the surface of live cells by confocal fluorescence microscopy using the assembly of Jo-In-GFP complexes. Live pneumococcal cells expressing CbpE-Jo or CbpE-In were incubated with exogenous His-GFP-In or PBS and after extensive washes, the fluorescence of the cells was quantified (see Methods). As shown in Fig. 7a, a fluorescence signal of about 550 arbitrary units (AU) was detected when cells expressing CbpE-Jo were incubated with exogenous His-GFP-In. A comparable fluorescence signal (about 450 AU) was observed with cells expressing a fusion between CbpE and sfGFP. By contrast, the fluorescence signal was decreased by more than 5-fold when cells expressing CbpE-Jo or CbpE-In were incubated with PBS or with His-GFP-In, respectively.

Since comparable fluorescence signals were measured with CbpE-Jo-GFP-In complex and sfGFP, we wondered whether BMW^JOIN^ would allow localizing proteins exposed at the surface of live pneumococcal cells. Cells were observed by epifluorescence microscopy and images were analyzed to define the subcellular localization of CbpE (Fig. 7b). Both labeling methods, using the GFP-derived BMW^JOIN^ tool or a direct fusion to sfGFP, show that CbpE in live cells displays a heterogeneous localization over the cell surface.

The procedure we have developed to detect proteins exposed at the pneumococcal surface using the BMW^JOIN^ tool is rapid (no preliminary sample treatment, limited washes steps or even no wash at all), adapted to live cells, highly specific since it relies on the Jo-In covalent association and restricted to surface proteins. By using the GFP-In version, we were also able to localize CbpE by fluorescence microscopy. The fluorescence intensity and the localization pattern were comparable to the configuration where CbpE was fused to sfGFP, making the BMW^JOIN^ procedure a very powerful tool to investigate the dynamic localization of proteins exposed at the surface of live cells. Applications of the BMW tool to detect and/or localize proteins expressed at the surface of other unencapsulated or encapsulated pneumococcal strains of *S. pneumoniae* will be performed in the future. Capsule should not impede the Jo/In proteins to have access to their partner exposed in the underlying spaces (proteins associated to the cell wall or to the membrane). Indeed, we and other groups have detected surface proteins in encapsulated pneumococcal strains, indicating that IgG molecules can cross the capsule although their molecular mass of 150 kDa is far superior to the Jo or In proteins, 10 and 16 kDa, respectively. Work is in progress to extend this application to other bacterial species like in Gram-negative bacteria to evaluate the capacity of Jo and In protein to cross the outer membrane and to eukaryotic cells.

## Methods

### Plasmid construction and site-directed mutagenesis

The Jo and In sub-domains are the two halves of the *S. pneumoniae* RrgA D2 domain^8^ and correspond to residues 144–218 and 587–722, respectively. The *jo* and *in* genes were amplified using the chromosomal DNA of the *S. pneumoniae* TIGR4 strain, and individually cloned into the MCS1 (pBMW2) and MCS2 (pBMW3), respectively, of pETDuet vectors (Novagen), leading to an His-tagged Jo protein while In remained untagged. The *jo* and *in* genes were also inserted in the same pETDuet vector to allow co-expression of His-Jo and In (pBMW4). The *in* gene was also cloned in the MCS1 of the pACYCDuet vector (Novagen) to allow production of an His-tagged In protein (pBMW1). Point mutations were introduced by PCR-based site-directed mutagenesis and verified by DNA sequencing (Beckman Coulter Genomics, Genewiz) (pBMW5, pBMW6, pBMW7). Fusion of Jo and In sequences and insertion of a 7-residues peptide was performed from pBMW4 by site-directed mutagenesis (pBMW8).

The Choline-Binding Domain E (CbdE, residues 335–627) was fused to either Jo or In or to both proteins and cloned into pETDuet and pACYCDuet vectors, all proteins were His-tagged (pBMW9 to pBMW12).

The His-Jo-GPCR * ECL-In synthetic gene was ordered from GeneArt Gene Synthesis (Life Technologies) and cloned into a pET vector (pBMW13). The *gpcr* gene coding for the full-length GPCR fused to a His-tag at the C-terminal end was cloned in the pIVEX2.3 vector (5PRIME) (pBMW14).

The gene encoding the superfolder variant of GFP (sfGFP) was optimized for expression in *S. pneumonia*e (GeneArt, Invitrogen) and cloned at the 5′ end of the *in* gene in a His tagged protein expression vector (pADG16).

### Protein expression and purification

Strains BL21/DE3 STAR or RIL of *E. coli* were used for protein expression, which was induced in Luria Bertani medium with 0.5 mM IPTG (isopropyl β-D-thiogalactopyranoside) at 37 °C for 3 h for Jo, In, GFP-In and Hla-His proteins (the latter protein was used as a negative control in the Western blot showed in Fig. 4), at 15 °C overnight for Jo and In proteins fused to CbdE and at 20 °C overnight for the His-Jo-GPCR * ECL-In construct. Cells from 1-L culture were harvested by centrifugation, resuspended in 50 ml of 50 mM Tris pH 8.0, 200 mM NaCl, 20 mM imidazole and a protease inhibitor cocktail (Complete EDTA free, Sigma-Aldrich) and lysed by sonication for 2 min (2 sec ON and 8 sec OFF). The lysate was clarified by centrifugation (20 min at 39,191 × *g* at 4 °C) and loaded onto a 1-ml HisTrap^TM^ HP column (GE Healthcare). Proteins of interest were recovered in the flow-through fraction or eluted by steps of increasing concentrations of imidazole (60 mM, 100 mM and 300 mM). They were dialyzed and further purified by gel filtration chromatography.

After Ni-affinity purification, the His-Jo-GPCR * ECL-In fusion protein was concentrated to about 5 mg/ml using a 10 kDa Amicon Ultra concentrator, dialyzed against 20 mM Phosphate Buffer 150 mM NaCl and processed by Covalab (France) to immunize rabbits.

The full-length His-GPCR was produced in a 2-ml Continuous Exchange Cell Free (CECF) system for 24 h at 37 °C in the presence of Brij58 0.5% and 32 μg of DNA (Cell Free Integrated Structural Biology Grenoble Platform). Aggregates were removed by centrifugation. The supernatant was loaded onto a 1-ml HisTrap^TM^ HP column equilibrated with 50 mM HEPES pH 7.4, 100 mM NaCl, Brij58 0.05%. The His-GPCR protein was eluted with 350 mM imidazole and further purified by gel filtration chromatography.

Accurate Jo and In concentrations were determined by BCA assay since the lack of aromatic residues impairs precise concentration determination by measuring the absorbance at 280 nm.

### Covalent complex reconstitution *in vitro*

The individual Jo and In fusions, as well as the Jo-In complex were separated by SDS-PAGE using 12.5% or 15% polyacrylamide gels. Gels were stained with Coomassie blue and band intensities were quantified using a GelChemiDoc imager (BioRad) and Image J software.

To detect the formation of the covalent complex in the crude bacterial extracts, cells from 1-L culture were resuspended in 50 ml of 50 mM Tris pH 8.0, 200 mM NaCl (plus 20 mM imidazole when the protocol includes a Ni-affinity chromatography purification step) and a protease inhibitor cocktail (Complete EDTA free, Sigma-Aldrich). Cells were lyzed by sonication for 2 min (2 sec ON and 8 sec OFF). The lysate was clarified by centrifugation (20 min at 39,191 × *g* at 4 °C). Samples of 5 μl were loaded onto polyacrylamide gels to separate the individual Jo and In from the covalent complex. Bacterial cultures producing separately Jo and In were mixed before cell lysis and purification of the Jo-In complex.

To visualize the formation of the complex *in vitro*, 10 μM of purified wild-type or mutant Jo and In proteins were mixed and incubated at 20 °C for 30 min in 50 mM Tris pH 8.0, 200 mM NaCl. To stop the reaction, samples were heated in SDS loading buffer for 5 min at 100 °C. The kinetics of the reaction was determined by incubating 10 μM of Jo and In in 50 mM Tris pH 8.0, 200 mM NaCl at 20 °C. Samples were harvested at different times and frozen to stop the reaction before analysis by SDS-PAGE and Coomassie blue staining. A complex-reconstitution experiment was also performed during 60 min in different buffers to test the influence of the pH.

The intensities of the Coomassie blue stained bands corresponding to Jo, In and the complex His-Jo-His-In were quantified using the Image J software. Calculation of the proportion of the complex reconstitution was performed by dividing the intensity of the covalent complex by the sum of the intensities of all the bands in the lane, then multiplying by 100.

### Crystallization and data collection

Crystals of Jo.In covalent complex obtained by co-expression were obtained by hanging-drop vapor diffusion by mixing 1 μL of protein (23 mg/mL in 50 mM Tris pH 8.0, 150 mM NaCl) and 1 μL of reservoir (2 M ammonium sulfate, 4% isopropanol). Crystals were subsequently cryoprotected in mother liquor containing 20% (v/v) glycerol, mounted in loops, flash-frozen under liquid nitrogen and exposed to the X-ray under a cold nitrogen stream at 100 K. A diffraction data set was collected (Table 2) at the ESRF (European Synchrotron Radiation Facility) ID23EH2 beamline (Grenoble, France).

### Structure determination and refinement

Statistics on data collection and refinement are summarized in Table 2. Experimental set up of the beamline and data quality of the collected images were monitored with MxCuBE^25^,^26^ and ADXV^27^. Data was indexed and scaled with XDS program suite^28^. XDSGUI^29^ was used to perform data quality and resolution cutoff check-up^30^,^31^,^32^,^33^,^34^. The reduced reflections information data was imported in to the CCP4 program suite^35^. The structure was solved by Molecular Replacement method (MR) with PHASER^36^, using as a search model the partial structure of RrgA (PDB: 2WW8), residues 186–627. The MR solution structure was rebuilt *de-novo*, in order to remove bias from the model, as implemented in ARP/wARP^37^. The structure was completed (29–630) by cycles of manual model building with COOT^38^. Water molecules were added to the residual electron density map as implemented in ARP/wARP^37^. Cycles of restrained refinement employing NCS and TLS^39^,^40^ were performed with REFMAC^41^ as implemented in the CCP4 program suite. Several cycles of manual model building and refinement were performed until *R*_*work*_ and *R*_*free*_ converged^42^. Stereochemical verification was performed with MolProbity^43^ and PROCHECK^44^. The secondary structure assignment was verified with DSSP^45^. Figures were generated with PyMol (http://www.pymol.org). Final refined model coordinates were deposited at the Protein Data Bank (PDB, http://www.rcsb.org)^46^, ID code: 5MKC.

### Antibody production

The production of rabbit polyclonal antibodies was performed by Covalab (France). The immunization protocol lasted for 67 days and included four antigen injections and four bleeds. His-Jo-GPCR * ECL-In was purified by Ni-affinity chromatography and dialyzed in 20 mM phosphate buffer, 150 mM NaCl. Each antigen injection was made with sample of 200 μl containing 1.4 mg of purified His-Jo-GPCR * ECL-In. The final bleed (50 to 70 ml) was processed to purify IgG specifically directed against the GPCR * ECL.

### Antibody immunopurification

Immobilization of proteins containing primary amines was performed on CNBr-activated 4B resin following the manufacturer’s instructions (GE Healthcare). Briefly, 5 to 10 mg of protein in 0.1 M NaHCO3 pH 8.3, 0.5 M NaCl (coupling buffer) was mixed with 1 ml resin and incubated for 2 h at 20 °C. Flow-through was discarded and the resin was washed with coupling buffer. Saturation of free carboxyl groups was realized by incubation with 0.1 M Tris pH 8.0 for 1 h at 20 °C. Uncoupled proteins were eluted by sequential washes with high salt solutions of alternating pH: 0.1 M NaAc pH 4.0, 0.5 M NaCl and 0.1 M Tris pH 8.0, 0.5 M NaCl. A volume of 1 ml of serum was incubated with the antigen-coupled resin previously equilibrated in 0.1 M Tris pH 8.0, 0.1 M NaCl (immunopurification buffer). After extensive washes with immunopurification buffer, IgG were eluted by 0.1 M glycine pH 3.0. Fractions of 500 μl were collected in tubes containing 50 μl of 1 M Tris pH 8.0 to neutralize the pH.

IgG specifically directed against the GPCR * ECL sequence were immunopurified from the rabbit polyclonal serum in a two-step procedure. Firstly, His-Jo-In was coupled to a CNBr-activated resin according to the protocol described above and the resin was incubated with the total serum. Antibodies against the His moiety, the Jo and In proteins were retained by the His-Jo-In resin while the antibodies directed against the GPCR * ECL regions were recovered in the flow-through fraction together with the serum proteins. The second step consisted in coupling the His-Jo-GPCR * ECL-In fusion protein on a CNBr-activated resin which was subsequently incubated with the fractionated serum sample. Antibodies directed against the GPCR * ECL moiety were retained and eluted by acidic pH. This procedure allowed to purify 70 μg anti-GPCR * ECL antibodies from 2 ml of total serum.

### Immunoanalysis

Blotting was performed by Western bloting. Proteins were separated by SDS-PAGE and subsequently electrotransferred on nitrocellulose membrane. Incubations times of 1 h were successively performed using the anti-GPCR * ECL antibody (diluted 1:500) and the anti-rabbit horseradish peroxidase conjugated antibody (diluted 1:10,000) in PBS, 0.03% Tween 20, 5% fat-free milk before detection with a chemiluminescent substrate (Thermo-Scientific).

Solid-phase binding assays were performed to test the different batches of immuno-purified antibodies. White 96-well microtiter plates (Greiner Bio One) were coated with 0.1 μg to 1 μg of His-GPCR, His-Jo-In or BSA as a control in 100 μl of PBS at 4 °C overnight. Saturation was performed by adding 200 μl/well of PBS, 2% BSA for 1 h at 20 °C. Five washes were performed using 200 μl of PBS. The antibodies were diluted 1:1000 in PBS, 0.03% Tween 20, 0.2% BSA and incubated for 1 h at 20 °C. Five washes were performed using 200 μl of PBS, 0.03% Tween 20 before adding 100 μl/well horseradish peroxidase-conjugated anti-His antibody (Sigma) (1:1,000 dilution) in PBS, 0.03% Tween 20, 0.2% BSA for 1 h at 20 °C. Four washes with 200 μl of PBS, 0.03% Tween 20 were performed. ECL solution (Pierce) (100 μl) was added to each well, and chemiluminescence was measured using a multiwell luminescence reader (Fluostar Optima, BMG Labtech).

### Bacterial strains and plasmids

Tables of strains, plasmids, and oligonucleotide primers can be found in Table S1 in the Supplemental Material. The unencapsulated pneumococcal strain R800 was used. Allelic replacements were performed using the Janus method, a two-step procedure, based on a bicistronic *kan-rpsL* cassette called Janus^47^. This method avoids polar effects and allows expression of the fusion proteins at physiological levels. The *cbpE* or *ftsZ* loci were substituted for the Janus (*kan-rpsL*) cassette conferring resistance to kanamycin and dominant streptomycin sensitivity (intermediate strains). Then, recombinant PCR DNA fragments (see Table S1 for primers) comprising the *cbpE* or *ftsZ* gene fused to *jo*, *in* or *sfgfp* genes and flanked on each side by sequences homologous to the upstream and downstream regions of the *cbpE* or *ftsZ* loci were used to transform the intermediate strains. Resistance to streptomycin was used as the selection maker. PCR of the modified *cbpE* or *ftsZ* loci were performed to verify the insertion at the correct position in the chromosome and sequenced to check the absence of mutations.

### Pneumococcal culture

Growth conditions, media, and bacterial transformation protocols were described previously^48^. Briefly, liquid cultures of *S. pneumoniae* strains were grown at 37 °C in C medium supplemented with 0.5% yeast extract^49^ or in Todd Hewitt medium (TH; BD Sciences). For transformation, about 250 ng of DNA was added to cells treated with synthetic competence stimulating peptide 1 in TH pH 8.0 supplemented with 1 mM CaCl_2_. Cells were grown for 2 h at 37 °C, and transformants were selected on Columbia (BD Sciences) blood (4%) agar plates containing the appropriate antibiotics (streptomycin 400 μg/ml or kanamycin 300 μg/ml).

### Complex formation at the surface of pneumococcal strains

Pneumococcal cells expressing CbpE or FtsZ fused to Jo or In proteins were grown in TH medium and harvested at mid-exponential growth phase (OD_600nm_ 0.3–0.6). Cells were washed and resuspended in PBS. His-Jo, His-In or His-GFP-In purified proteins were added in the cell suspension at a final concentration of 0.1 mg/ml and incubated with cells at 20 °C for 1 h. Cells were washed, resuspended in PBS and aliquots were mixed with 1/4 volume of Laemmli buffer 4× (1× buffer composition: 0.1% 2-mercaptoethanol, 0.0005% Bromophenol blue, 10% glycerol, 2% SDS, 63 mM Tris pH 6.8) and boil for 10 min to allow cell lysis. Aliquots were loaded on BioRad Criterion 4–12% gels run in MOPS buffer. Immunodetections were performed with anti-CbpE, anti-FtsZ and anti-His (Sigma) antibodies

### Spinning disk confocal microscopy image acquisition and analysis

Cells were grown at 37 °C in TH to an OD_600nm_ of 0.3, transferred to microscope slides, and observed at 37 °C on an Olympus IX81 microscope equipped with a Yokogawa CSU-X1 confocal spinning disk unit. Image acquisition was performed using a UAPON 100× (N.A. 1.49) oil immersion objective and Andor^TM^iXon Ultra Electron Multiplying Charge Coupled Device (EMCCD) camera. Epifluorescence confocal of GFP signal (488 nm excitation and 520/28 nm emission) and Differential Interference Contrast (DIC) transmission imaging were collected using IQ software (Andor^TM^), and further analyzed using Volocity (Perkin Elmer^TM^). In addition to the specific GFP channel, images were acquired in the red emission channel (561 nm excitation and 617/73 nm emission) to estimate the background autofluorescence signal. In each frame (total of five) and for each cell, the maximal fluorescence intensities were measured and the mean maximal intensity of GFP for each sample was calculated after substraction of the autofluorescence levels. The error bars correspond to the standard deviation of the analysis of the five independent frames acquired for each sample.

## Additional Information

**How to cite this article:** Bonnet, J. *et al*. Autocatalytic association of proteins by covalent bond formation : a Bio Molecular Welding toolbox derived from a bacterial adhesin. *Sci. Rep.* **7**, 43564; doi: 10.1038/srep43564 (2017).

**Publisher's note:** Springer Nature remains neutral with regard to jurisdictional claims in published maps and institutional affiliations.

## Supplementary Material

Supplementary Information

## Figures and Tables

**Table 1:** Molecular masses of Jo and In partners determined by ESI-MS.

	*M*_average_^a^ (Da)		Δ (*M*_expected_ - *M*_observed_)^b^ (Da)	NH_3_ units lost
	Expected^b^	Observed by ESI-TOF		
His-Jo	10 470	10 471	0	0
His-In	16 318	16 319	0	0
His-Jo. His-In	26 770	26 772	18	1
His-Jo_K191A_	10 413	10 413	0	0
His-In_D600A_	16 274	16 274	0	0
His-In_N695A_	16 275	16 275	0	0
His-Jo-GSTPGSV-In	25 874	25 857	17	1

^a^Average molecular mass.

^b^The values of the expected masses take into account the formation of the isopeptide bond.

**Table 2:** Data collection, molecular replacement and structure refinement statistics.

**DATA COLLECTION**	
X-ray source	ID23EH2
Detector	MarMosaic 225
Wavelength (Å)	0.87260
Scan-range (°)	150
Oscillation (°)	1
Space group	P2_1_2_1_2_1_
*a* (Å)	132.54
*b* (Å)	134.57
*c* (Å)	144.47
Mosaicity (°)	0.214
Overall resolution (Å)	45.91–2.04
No. observed/unique reflections	689197/153725
High resolution shell (Å)	2.17–2.04
Completeness (%) (last shell)	93.6 (83.8)
*R*_*sym*_ (last shell)	4.6 (54.9)
*I*/*σ(I*) (last shell)	30.60 (3.02)
Wilson plot B-factor (Å^2^)	41.71
**MOLECULAR REPLACEMENT**	
Solvent content (%)	40
Mol/ASU	6
Phaser RZF/TZF scores	8/26, 9/50, 8/60, 7/72, 8/68, 10/79
Phaser LLG scores	481, 1837, 3768, 6450, 9539, 13378, 15960
**REFINEMENT**	
Initial *R*_*work*_/*R*_*free*_ (%)	27.44/31.56
Final *R*_*work*_/*R*_*free*_ (%)	21.32/24.31
RMS deviation, bond lengths (Å)	0.009
RMS deviation, bond angles (°)	1.266
Mean B-factor (Å^2^)	53.73
No. of protein atoms	10949
No. of water molecules	1023
No. of sulfate molecules	15
No. of ions (Ni, Ca)	3, 3
Residues in most favored/allowed region of Ramachandran plot (%)	100.0
