# Supplementary material for: Autocatalytic association of proteins by covalent bond formation: a Bio Molecular Welding toolbox derived from a bacterial adhesin
Source: Sci Rep. 2017 Mar 2;7:43564. doi: 10.1038/srep43564 (PMC5333627; doi:10.1038/srep43564)
Supplement: Supplementary Information [file srep43564-s1.pdf]

Autocatalytic association of proteins by covalent bond formation : a Bio Molecular Welding toolbox derived from a bacterial adhesin

Bonnet J.[1], Cartannaz J.[1], Tourcier G.[2], Contreras-Martel C.[1], Kleman J.P.[1], Morlot C.[1], Vernet T.[1*], and Di Guilmi A.M.[1*]

Figure S1: RrgA, Jo and In sequences. Lys191 (Jo sequence), Asp600 (In sequence) and Asn695 (In sequence) residues involved in the formation of the isopeptide bond are highlighted in grey.

RrgA (Jo and In sequences are underlined)

MLNRETHMKKVRKIFQKAVAGLCCISQLTAFSSIVALAETPETSPAIGKVVIKETGEGGALLGDAVFELKN
NTDGTTVSQRTEAQTGEAIFSNIKPGTYTLTEAQPPVGYKPSTKQWTVEVEKNGRTTVQGEQVENREEALS
DQYPQTGTYPDVQTPYQIIKVDGSEKNGQHKALNPNPYERVIPEGTLSKRIYQVNNLDDNQYGIELTVSGK
TVYEQKDKSVPLDVVILLDNSNSMSNIRNKNARRAERAGEATRSLIDKITSDSENRVALVTYASTIFDGTE
FTVEKGVADKNGKRLNDSLFWNYDQTSFTTNTKDYSYLKLTNDKNDIVELKNKVPTEAEDHDGNRLMYQFG
ATFTQKALMKADEILTQQARQNSQKVIFHITDGVPTMSYPINFNHATFAPSYQNQLNAFFSKSPNKDGILL
SDFITQATSGEHTIVRGDGQSYQMFTDKTVYEKGAPAAFPVKPEKYSEMKAAGYAVIGDPINGGYIWLNWR
ESILAYPFNSNTAKITNHGDPTRWYYNGNIAPDGYDVFTVGIGINGDPGTDEATATSFMQSISSKPENYTN
VTDTTKILEQLNRYFHTIVTEKKSIENGTITDPMGELIDLQLGTDGRFDPADYTLTANDGSRLENGQAVGG
PQNDGGLLKNAKVLYDTTEKRIRVTGLYLGTDEKVTLTYNVRLNDEFVSNKFYDTNGRTTLHPKEVEQNTV
RDFPIPKIRDVRKYPEITISKEKKLGDIEFIKVNKNDKKPLRGAVFSLQKQHPDYPDIYGAIDQNGTYQNV
RTGEDGKLTFKNLSDGKYRLFENSEPAGYKPVQNKPIVAFQIVNGEVRDVTSIVPQDIPAGYEFTNDKHYI
TNEPIPPKREYPRTGGIGMLPFYLIGCMMMGGVLLYTRKHP

Jo : residues 142 to 220 in RrgA sequence (underlined)

SDQYPQTGTYPDVQTPYQIIKVDGSEKNGQHKALNPNPYERVIPEGTLSKRIYQVNNLDDNQYGIELTVSG
KTVYEQKD

TCTGACCAGTATCCACAAACAGGGACTTATCCAGATGTTCAAACACCTTATCAGATTATTAAGGTAGATGG
TTCGGAAAAAAACGGACAGCACAAGGCGTTGAATCCGAATCCATATGAACGTGTGATTCCAGAAGGTACAC
TTTCAAAGAGAATTTATCAAGTGAATAATTTGGATGATAACCAATATGGAATCGAATTGACGGTTAGTGGG
AAAACAGTGTATGAACAAAAAGAT

In : residues 588 to 722 in RrgA sequence (underlined)

TEKKSIENGTITDPMGELIDLQLGTDGRFDPADYTLTANDGSRLENGQAVGGPQNDGGLLKNAKVLYDTTE
KRIRVTGLYLGTDEKVTLTYNVRLNDEFVSNKFYDTNGRTTLHPKEVEQNTVRDFPIPKIRDVR

ACTGAAAAGAAATCAATTGAGAATGGTACGATTACAGATCCGATGGGTGAGTTAATTGATTTGCAATTGGG
CACAGATGGAAGATTTGATCCAGCAGATTACACTTTAACTGCAAACGATGGTAGTCGCTTGGAGAATGGAC
AAGCTGTAGGTGGTCCACAAAATGATGGTGGTTTGTTAAAAAATGCAAAAGTGCTCTATGATACGACTGAG
AAAAGGATTCGTGTAACAGGTCTGTACCTTGGAACGGATGAAAAAGTTACGTTGACCTACAATGTTCGTTT
GAATGATGAGTTTGTAAGCAATAAATTTTATGATACCAATGGTCGAACAACCTTACATCCTAAGGAAGTAG
AACAGAACACAGTGCGCGACTTCCCGATTCCTAAGATTCGTGATGTGCGG

Table S1: Strains, Plasmids and Oligonucleotides

Construct	Genotype/description/sequence	Source
***S. pneumoniae* strains**		
R800	*R6 rpsL1 ; Str^R*	1
CbpE-Jo	*R800 rpsL1 ; cbpE::cbpE-jo; Str^R*	This work
CbpE-In	*R800 rpsL1 ; cbpE::cbpE-in; Str^R*	This work
CbpE-sfGFP	*R800 rpsL1 ; cbpE::cbpE-sfgfp; Str^R*	This work
FtsZ-Jo	*R800 rpsL1 ; ftsZ::ftsZ-jo; Str^R*	This work
FtsZ-In	*R800 rpsL1 ; ftsZ::ftsZ-in; Str^R*	This work
**Plasmids**		
pBMW1	*His6-In* (*pACYCDuet*)	This work
pBMW2	*His6-Jo* (*pETDuet*)	This work
pBMW3	*In* (*pETDuet*)	This work
pBMW4	*His6-Jo + In* (*pETDuet*)	This work
pBMW5	*His6-In^{D600A}* (*pACYCDuet*)	This work
pBMW6	*His6-Jo^{K191A}* (*pETDuet*)	This work
pBMW7	*His6-In^{N695A}* (*pACYCDuet*)	This work
pBMW8	*His6-Jo*-GSTPGSV-*In* (*pETDuet*)	This work
pBMW9	*His6-Jo-CbdE* (*pETDuet*)	This work
pBMW10	*His6-In-CbdE* (*pACYCDuet*)	This work
pBMW11	*His6-Jo-CbdE-Jo* (*pETDuet*)	This work
pBMW12	*His6-Jo-CbdE-In* (*pETDuet*)	This work
pBMW13	*His6-Jo-GPCR*ECL-In (pETDuet)*	This work
pBMW14	*His6-GPCR (pIVEX2.3)*	This work
pADG16	*His8-sfGFP-In (pET vector)*	This work
**Oligonucleotides**		
FORpBMW1	CGCGGATCCGACTGAAAAGAAATCAATTGAGAATGG	This work
REVpBMW1	GCGCTCGAGTCAATCACGAATCTTAGGAATCGGGAAGTCC	This work
FORpBMW2	CGCGGATCCGTCTGACCAGTATCCACAAACAGGG	This work
REVpBMW2	GCGGTCGACTCAATCTTTTTGTTCATACACTGTTTTCCC	This work
FORpBMW3	CGCAGATCTGACTGAAAAGAAATCAATTGAGAATGG	This work
REVpBMW3	GCGCTCGAGTCAATCACGAATCTTAGGAATCGGGAAGTCC	This work
FORpBMW5	TCAATTGAGAATGGTACGATTACAGCTCCGATGGGTGAGTTAATTGATTTGC	This work
REVpBMW5	GCAAATCAATTAACTCACCCATCGGAGCTGTAATCGTACCATTCTCAATTGA	This work
FORpBMW6	GTGATTCCAGAAGGTACACTTTCAGCGAGAATTTATCAAGTGAATAATTTGG	This work
REVpBMW6	CCAAATTATTCACTTGATAAATTCTCGCTGAAAGTGTACCTTCTGGAATCAC	This work
FORpBMW7	GTAAGCAATAAATTTTATGATACCGCTGGTCGAACAACCTTACATCCTAAGG	This work
REVpBMW7	CCTTAGGATGTAAGGTTGTTCGACCAGCGGTATCATAAAATTTATTGCTTAC	This work
FORpBMW8	GGGAAAACAGTGTATGAACAAAAAGATGGTTCTACCCCGGGTTCTGTTACTGAAAAGAAA TCAATTGAGAATGG	This work
REVpBMW8	CCATTCTCAATTGATTTCTTTTCAGTAACAGAACCCGGGGTAGAACCATCTTTTTGTTCATA CACTGTTTTCCC	This work
FORpBMW9-1	GGAAAACAGTGTATGAACAAAAAGATCTGCAGGTCGACAAGCTTGCGGCCGCATAATGCT TAAG	This work
REVpBMW9-1	CTTAAGCATTATGCGGCCGCAAGCTTGTCGACCTGCAGATCTTTTTGTTCATACACTGTTTT CC	This work
FORpBMW9-2	CGCCTGCAGCAAGCTGGTTGGCATAAGAGTGCATATGGG	This work
REVpBMW9-2	GCGGTCGACCTGTTCTGATTCCGATTTGTTTACACTCG	This work
FORpBMW10-1	CGACTTCCCGATTCCTAAGATTCGTGATCTGCAGGTCGACAAGCTTGCGGCCGCATAATGC TTAAGTCG	This work
REVpBMW10-1	CGACTTAAGCATTATGCGGCCGCAAGCTTGTCGACCTGCAGATCACGAATCTTAGGAATCG GGAAGTCG	This work
FORpBMW11	CGCGTCGACAAGCTTTCTGACCAGTATCCACAAACAGGGAC	This work
REVpBMW11	GCGGCGGCCGCTTAATCTTTTTGTTCATACACTGTTTTCCC	This work
FORpBMW12	CGCGTCGACAAGCTTACTGAAAAGAAATCAATTGAGAATGGTACG	This work
REVpBMW12	GCGGCGGCCGCTTAATCACGAATCTTAGGAATCGGGAAGTCGC	This work
FORpBMW14	CGCGCGGCCGCAATAGCTTTAACTATACCACACCG	This work
REVpBMW14	GCGCTCGAGAACTGCCTGTGTTTTCTGTGC	This work
pADG16For	CGCAAGCTTACTGAAAAGAAATCAATTGAGAATGG	This work
pADG16Rev	GCGCTCGAGTTATTAATCACGAATCTTAGGAATCGGGAAG	This work
FORJanus	CCGTTTGATTTTTAATGGATAATG	2
REVJanus	AGAGACCTGGGCCCCTTTCC	2
FOR1CbpE	CGCAGCCAGCAAAGACTATG	This work
REV1CbpE	CCATTAAAAATCAAACGGCTGTTCTGATTCCGATTTG	This work
FOR2CbpEGFP	GGAAAGGGGCCCAGGTCTCTGAAAAGAAGGTTTTAGGGCC	This work
REV2CbpEGFP	CCAAGTGATTTTCCACTTTCC	This work
REV3CbpEGFP	CCTTTAGAACCGGTCGTACGCTGTTCTGATTCCGATTTG	This work

FOR4CbpEGFP	CAAATCGGAATCAGAACAGCGTACGACCGGTTCTAAAGG	This work
REV4CbpEGFP	GGCCCTAAAACCTTCTTTTCTTATTATTTGTACAATTCATCC	This work
FOR5CbpEGFP	GGATGAATTGTACAAATAATAAGAAAAGAAGGTTTTAGGGCC	This work
REV5CbpEGFP	GATAAGTTCTGAACCGGTCGTACGCTGTTCTGATTCCGATTTG	This work
FOR6CbpEGFP	CAAATCGGAATCAGAACAGCGTACGACCGGTTCAGAACTTATC	This work
REV6CbpEGFP	GGCCCTAAAACCTTCTTTTCTTATTAACGGTGTCCCAATTTACTAGG	This work
FOR7CbpEGFP	CCTAGTAAATTGGGACACCGTTAATAAGAAAAGAAGGTTTTAGGGCC	This work
FORJo1	TCTGACCAGTATCCACAAACA	This work
REVJo2	TTAATCTTTTTGTTCATACACTGTTTTC	This work
REVCbpEJoJB23	GTTTGTGGATACTGGTCAGACTGTTCTGATTCCGATTTGT	This work
FORCbpEJoJB24	TGTATGAACAAAAAGATTAATAGAAAAGAAGGTTTTAGGGC	This work
FORIn1	ACTGAAAAGAAATCAATTGAGAATG	This work
REVIn2	TTAATCACGAATCTTAGGAATCGG	This work
REVCbpEInJB25	TCAATTGATTTCTTTTCAGTCTGTTCTGATTCCGATTTGT	This work
FORCbpEInJB26	TTCCTAAGATTCGTGATTAATAGAAAAGAAGGTTTTAGGGC	This work
FORFtsJoMJ42	CCTATCCGCCTCTTGCAAGC	This work
REVFtsJoMJ47	GTAGGAGAGAACCATGTCTTTAAAAG	This work
REVFtsJoJB15	GTTTGTGGATACTGGTCAGAACGATTTTTGAAAAATGGAGGT	This work
FORFtsJoJB16	TGTATGAACAAAAAGATTAACATTTTTCAAAAATCGTTAAGTAAAT	This work
FORFtsZInMJ42	CCTATCCGCCTCTTGCAAGC	This work
REVFtsZInMJ47	GTAGGAGAGAACCATGTCTTTAAAAG	This work
REVFtsZInJB17	TCAATTGATTTCTTTTCAGTACGATTTTTGAAAAATGGAGGT	This work
FORFtsZInJB18	TTCCTAAGATTCGTGATTAACATTTTTCAAAAATCGTTAAGTAAAT	This work

1. Lefevre, J. C., Claverys, J. P. & Sicard, A. M. Donor deoxyribonucleic acid length and marker effect in pneumococcal transformation. *J. Bacteriol.* **138**, 80-86 (1979).

2. Sung, C. K., Li, H., Claverys, J. P. & Morrison, D. A. An *rpsL* cassette, *Janus*, for gene replacement through negative selection in *Streptococcus pneumoniae*. *Appl. Environ. Microbiol.* **67**, 5190-5196 (2001).
